# Rapid diagnosis of non-tuberculous mycobacterial pulmonary diseases by metagenomic next-generation sequencing in non-referral hospitals

**DOI:** 10.3389/fcimb.2022.1083497

**Published:** 2023-01-24

**Authors:** Jing Wang, Huan Xu, Xi Wang, Jian Lan

**Affiliations:** ^1^ Department of Respiratory and Critical Care Medicine, the Second Clinical Hospital of Chongqing Medical University, Chongqing, China; ^2^ Department of Scientific Affairs, Vision Medicals Center for Infection Diseases, Guangzhou, China

**Keywords:** non-tuberculous mycobacteria (NTM), pulmonary disease, metagenomic next-generation sequencing (mNGS), rapid diagnosis, earlier targeted treatment

## Abstract

**Objectives:**

The incidence of non-tuberculous mycobacterial pulmonary disease (NTM-PD) has increased steadily globally, but the current culture-based diagnosis of NTM-PD is difficult and time-consuming, leading to a high possibility of misdiagnosis. Therefore, new methods should be introduced to improve the processes for clinical diagnosis of this disease.

**Methods:**

Our retrospective observational study enrolled 12 NTM-PD patients who were identified by way of metagenomic next-generation sequencing (mNGS), as well as the characteristic radiological presentation of slowly progressed, usually concomitant bronchiectasis, small cavitary opacity, and multiple nodules that respond poorly to empirical antibiotic therapy. These patients received the recommended drug regimen based on the identified non-tuberculous mycobacteria (NTM) species. Clinical data, including symptoms, laboratory tests, dynamic computed tomography imaging, treatment, and outcome, were recorded and analyzed.

**Results:**

The results of mNGS were all positive, with the standard specifically mapped read numbers (SDSMRN) of NTM ranging from 1 to 766; this was confirmed in six patients via quantitative polymerase chain reaction (qPCR) analysis. The duration fromsample collection tomNGS results was 1–4 days. Among our 12 patients (except for one lost to follow-up) the CT imaging for 11 patients showed significant absorption of lesions.

**Conclusions:**

Our results draw attention to NTM infection as a possible cause of community-acquired pneumonia, especially in patients with suggestive radiological presentation and poor responses to empirical antibiotic therapy. Our study also indicated that mNGS represented a potentially effective tool for the rapid identification of NTM in the respiratory sample. Improved clinician awareness combined with the utilization of mNGS could guide earlier diagnosis and targeted treatment, and finally improved the prognoses of patients with NTM-PD.

## Introduction

The incidence of morbidity and mortality owing to non-tuberculous mycobacterial pulmonary diseases (NTM-PD) has increased worldwide and is rapidly becoming a public health problem (Kumar and Loebinger, 2021; Tan et al., 2021). The treatment of non-tuberculous mycobacterial (NTM) pulmonary diseases is particularly challenging as NTM represents over 190 species and subspecies of *Mycobacterium*, other than *M. tuberculosis* and *M. leprae*, and treatment regimens vary for different species (Daley et al., 2020a). Thus, early diagnosis and species detection is very important to guide the clinical targeted treatment of NTM-PD.

Non-tuberculous mycobacteria are ubiquitous in the environment and are generally considered as being of low pathogenicity to humans who have normal immune defenses (Ratnatunga et al., 2020). Increased susceptibility to infection was seen in patients with underlying structural pulmonary diseases, such as cystic fibrosis, chronic obstructive pulmonary disease (COPD), bronchiectasis, or with immunocompromised states (e.g., patients with AIDS, or having had solid organ transplants, or being treated with corticosteroids or other immunosuppressants) (Griffith et al., 2007). In addition, a lower body mass index (BMI) is common among patients with NTM-PD, and is associated with a worse prognosis (Song et al., 2021)

The symptoms of NTM-PD are non-specific and include cough, sputum production, breathlessness, fatigue, weight loss, and fever. NTM infections are typically chronic and slowly progressive. The disease is insidious and it mimics others, which can lead to a delayed diagnosis for patients with NTM-PD. Typical radiographic findings from NTM-PD patients include fibrocavitary, nodules, and bronchiectasis. Patients usually have combined radiological abnormalities, and are subclassified into bronchiectasis with nodules (i.e., “nodular bronchiectatic” phenotype) or cavitation with fibrosis (i.e., “fibrocavitary” phenotype) (Cowman et al., 2019).

The diagnosis of NTM-PD is made based on a combination of clinical, radiographic, and microbiological data (Cowman et al., 2019; Daley et al., 2020a), with the culture of respiratory samples remaining the gold standard for laboratory confirmation of NTM-PD (Daley et al., 2020c). However, the culture-based diagnosis of NTM-PD is difficult and time-consuming. It has been recommended that bacterial culturing is undertaken for at least 8 weeks (Haworth et al., 2017). In addition, the optimal growth temperature differs for NTM species, and as all NTM are fastidious organisms, some, like *M. tilburgii*, have yet to be successfully cultured (van Ingen, 2013). Furthermore, culturing is less applicable with a patients with nodular bronchiectasis because of the low bacterial burden (van Ingen, 2015). Most importantly, because of the lack of clinician awareness of NTM-PD and the poor accessibility to laboratory resources for mycobacterial culture and molecular methods for identification or speciation, the diagnosis of NTM-PD is rarely made in non-referral hospitals. In these settings, unless a predefined suspicious pathogen, a negative culture result of lower respiratory secretions will be reported if growth was not detected within 48 to 72 hours of incubation (York and Gilligan, 2004). Collectively, the culture-based diagnosis of NTM-PD poses great challenges, and the development of alternative, rapid, universally available, and hypothesis-free diagnostic approaches is urgently required.

Metagenomic next-generation sequencing (mNGS) has the potential to detect nearly all pathogens in a clinical sample (Langelier et al., 2018; Chen et al., 2021). Thus, culture-independent and unbiased mNGS has been widely used in investigations of the etiology of infectious diseases (Chiu and Miller, 2019; Gu et al., 2021), and has an obvious advantage in the diagnosis of uncommon respiratory pathogens, including NTM. In addition, the sensitivity of mNGS in NTM identification is higher than that of bacterial culturing (Shi et al., 2020). However, to our knowledge, there have been no studies that systematically evaluate the clinical performance of mNGS in the diagnosis of NTM-PD until now. In this study, we retrospectively investigated the effectiveness of mNGS in identifying NTM-PD. Our study has significant implications for improving clinician awareness and early diagnosis rates of NTM-PD.

## Materials and methods

### Study design

We conducted a retrospective study of adult inpatients between July 2020 and January 2022 from the Second Clinical Hospital of Chongqing Medical University, a non-referral, tertiary teaching hospital in Chongqing, China. The diagnosis of NTM-PD was made according to the Chinese guidelines for the diagnosis of non-tuberculous mycobacterial diseases in 2021 to 2020 (Chinese Society of Tuberculosis, 2021). According to these guidelines, the patient can be diagnosed with NTM-PD if they meet the required clinical and radiological criteria that cannot be explained by other diagnoses, as well as one of the relevant microbiological criteria. The specific criteria are as follows: (1) pulmonary or systemic symptoms; and (2) nodular or cavitary opacities on a chest radiograph, or a high-resolution computed tomography scan that shows bronchiectasis with multiple small nodules. Patients also had to meet any one of the following microbiological criteria: (A) positive culture results and/or positive NTM molecular biology detection from at least two separate expectorated sputum samples; (B) positive culture results and/or positive NTM molecular biology detection from at least one bronchial wash or lavage; (C) transbronchial or other lung biopsy results with mycobacterial histological features (i.e., granulomatous inflammation or acid-fast bacilli), and positive culture results and/or positive NTM molecular biology detection from a lung biopsy; (D) transbronchial or other lung biopsy results with mycobacterial histological features (i.e., granulomatous inflammation or acid-fast bacilli); and (E) at least one positive culture result and/or positive NTM molecular biology detection from a sputum sample, bronchial wash, or lavage. Based on these guidelines, our criteria for inclusion were as follows: patients were aged between 18 and 80 years, (1) with pulmonary or systemic symptoms, such as fever, fatigue, cough, expectoration; (2) with nodular or cavitary opacities or bronchiectasis with multiple small nodules, as detected on a high-resolution computed tomographic scan, which progress slowly and usually appear concomitantly; (3) who responded poorly to an initial empiric antibiotic therapy; (4) with positive NTM molecular biology detection from at least one bronchoalveolar lavage fluid (BALF) or sputum sample; and (5) who had a positive response to targeted anti-NTM treatment. Furthermore, we performed fluorescence quantitative polymerase chain reaction (qPCR) analysis using a Applied Biosystems 7,500 real-time PCR system (ABI) to validate the mNGS results. Written informed consent was obtained from all patients or their relatives. This study was approved by the Ethics Committee of the Second Clinical Hospital of Chongqing Medical University [reference number: 2021(104)] and conducted in accordance with the Declaration of Helsinki.

### Data collection

Baseline characteristics (including age, sex, underlying samples, such as BALF, sputum specimens, and blood, diseases, medication use history, and epidemiology history), Pneumonia Severity Index (PSI) score on admission, laboratory test results (including routine blood and biochemical tests), imaging findings, and antibiotic treatment, were extracted from electronic medical records. Additional follow-up data on treatments and outcomes were also collected.

### Conventional microbiological tests

Routine samples, such as BALF, sputum specimens, and blood, were collected. Conventional microbiological tests were performed on admission, including sputum and BALF culture and smear (acid-fast staining for *M. tuberculosis*), the respiratory pathogen panel by PCR analysis (for the detection of 13 respiratory pathogens), the respiratory pathogen panel by immunological test (for the detection of nine respiratory pathogens), the serum tuberculosis antibody test (TB-Ab IgM, TB-Ab IgG), serum antigen test (1,3-β-D-glucan antigen), the serum and BALF galactomannan test (*Aspergillus* spp.), the serum interferon gamma release assay (*M. tuberculosis*), the tuberculosis skin test (PPD skin test), and the BALF GeneXpert assay (*M. tuberculosis*).

The thirteen respiratory pathogens detected by multiplex PCR were: *Streptococcus pneumoniae*, *Staphylococcus aureus*, meticillin-resistant *Staphylococcus*, *Escherichia coli*, *Klebsiella pneumoniae*, *Pseudomonas aeruginosa*, *Acinetobacter baumannii*, *Stenotrophomonas maltophilia*, *Haemophilus influenzae*, *Mycoplasma pneumoniae*, *Chlamydia pneumoniae*, *Legionella pneumophila*, and the *M. tuberculosis complex*. The nine respiratory pathogens detected by immunological testing were: *Legionella pneumophila*, *M. pneumoniae*, *Coxiella burnetii*, *C. pneumoniae*, respiratory syncytial virus, adenovirus, influenza A virus, influenza B virus, and parainfluenza virus.

### Metagenomics next-generation sequencing

Samples of BALF (*n* = 11) and sputum (*n *= 1) were collected and transported to mNGS laboratories for sequencing following standard procedures (https://emergency.cdc.gov). DNA was extracted using the TIANamp Micro DNA Kit (TIANGEN BIOTECH, Beijing, China) following the manufacturer’s instructions. Human DNA was removed using Benzonase (Qiagen) and Tween 20 (Sigma-Aldrich). DNA libraries were constructed through DNA fragmentation, end-repair, adapter-ligation, and PCR amplification using a Nextera XT DNA Library Prep Kit (Illumina, San Diego, CA) or according to the standard protocol of the BGISEQ-500 sequencing platform and Ion Torrent (PGM) platform. The libraries were assessed by an Agilent 2,100 Bioanalyzer (Agilent Technologies, Santa Clara, CA, United States), and quantified using a Qubit dsDNA HS Assay Kit (Thermo Fisher Scientific, MA, United States). Pooled libraries were sequenced on a NextSeq Dx550 sequencer (Illumina, CA, United States), or on a BGISEQ-500 sequencer (BGI, Shenzhen, China), or on an Ion Torrent (PGM) platform (Thermo Fisher Scientific). The sequencing data were trimmed by the removal of low-quality reads and short reads, low-complexity reads, and adapters. The human host sequences were aligned to the human reference genome (GRCh38) using Burrows–Wheeler alignment (BWA). Subsequently, the remaining microbial sequences were classified by simultaneously aligning to four Microbial Genome Databases, consisting of bacteria, viruses, fungi, and parasites. The curated microbial databases were downloaded and optimized from public database, such as Biotechnology Information Reference Sequence (RefSeq) database (release version 68).

### Criteria for a positive mNGS result for non-tuberculous mycobacteria

The species-specific read number (SSRN) was also described as specifically mapped read number (SMRN). In this study, SMRN was normalized to 20 million (M) of the total number of sequencing reads, which was called ‘standard SMRN’ (SDSMRN). The equation for the SDSMRN was as follows: SDSMRN = SMRN × 20 million/total sequencing reads. Non-tuberculous mycobacteria were considered detected if: (1) its genus was among the top 20 with the highest SDSMRN; (2) it was ranked first within its genus; and (3) it had a SDSMRN of > 1 (Qian et al., 2020).

### qPCR detection of non-tuberculous mycobacteria

To validate the mNGS results, we performed fluorescence quantitative polymerase chain reaction (qPCR) analysis on an Applied Biosystems 7,500 real-time PCR system (ABI). **Table 1** primer sequences used for qPCR analysis of selected NTM species.

**Table 1:** The amplification primers for NTM are listed below:

Species of NTM	Forward primer (5′–3′)	Reverse primer (5′–3′)
*M. kansasii*	GCGGAGCTTGCCTATACATTTG	AGGTCCGTACAGTTCCATCTC
*M. avium*	AAATGCGCTCGTACCGAATC	CCGTACAGCAGGATCAACAC
*M. intracellulare*	GTTGGCGATCACTCGCTACT	CCGAACCCATCCACGTTTCC
*M. abscessus*	TGTCGTCTGTGCATCAACTC	CCAATGCTGGACCCAAACAC
*M. fortuitum*	AGCCAGCCACCACGAATATC	GGCTGAGGAACTCCGAACAC

## Results

### Clinical *features and laboratory examinations* of the participants

A total of 1362 patients in our hospital received a mNGS analysis. A total of 12 (0.88%) patients, who were suggestively diagnosed with NTM-PD as a result of mNGS analysis, were retrospectively evaluated in our study. The demographic and clinical characteristics of these patients are shown in **Table 2**. Among them, five were male and seven were female, with a median age of 60 years (age ranged from 26 to 75 years; interquartile range 34.75–66.75 years) and body mass index (BMI) of 19.96 kg/m^2^ (interquartile range 18.50–21.53 kg/m^2^). In total, eight patients had at least one underlying illness (e.g., COPD or coronary heart disease), while only one patient (i.e., patient 2) had a history of bronchiectasis. The duration of illness from onset to admission varied largely, ranging from 1 day to over 3 years, with a median of 20 days. The usual respiratory symptoms of these patients were coughing, sputum production, and dyspnea. Systemic symptoms, such as fatigue, chills, and low-grade fever, were also common. One patient presented with symptoms associated with the location of lesion (i.e., patient 8). The PSI score was calculated for 12 patients, with a medium score of 50.5 (interquartile range, 26–60.25), and only one patient was scored in risk class 4.

**Table 2:** Baseline characteristics, clinical manifestation, and radiographic findings of patients on admission.

Characteristics on admission	Patients	Normal range
Male sex, *n* (%)	5/12 (41.67%)	
Age (years), median value (range)	60 (34.75–66.75)	
BMI (kg/m^2^), median value (range)	19.96 (18.50–21.53)	18.5–23.9
Underlying conditions, *n* (%)		
Bronchiectasis	1/12 (8.33%)	
Asthma	1/12 (8.33%)	
COPD	3/12 (25%)	
Diabetes mellitus	1/12 (8.33%)	
Hypertension	2/12 (16.67%)	
Coronary heart disease	3/12 (25%)	
Dermatomyositis	1/12 (8.33%)	
Duration from illness onset to admission (days), median value (range)	20 (14.50–60)	
Currently a smoker, *n* (%)	4/12 (33.33%)	
Currently an alcoholic, *n* (%)	3/12 (25%)	
Clinical manifestations, *n* (%)		
Fever > 38.5°C	1/12 (8.33%)	
Cough	6/12 (50%)	
Expectoration	6/12 (50%)	
Hemoptysis	2/12 (16.67%)	
Dyspnea	4/12 (33.33%)	
Palpitation	1/12 (8.33%)	
Shiver	1/12 (8.33%)	
Fatigue	1/12 (8.33%)	
Chest tightness	1/12 (8.33%)	
Abdominal pain	1/12 (8.33%)	
Chest pain	1/12 (8.33%)	
Weight loss	1/12 (8.33%)	
Heart rate (beats per min), median value (range)	90 (83.75–100)	60–100
Respiration (breaths per min), median value (range)	20 (20–20.25)	12–20
Pneumonia Severity Index (PSI)		
Median (interquartile range)	50.5 (26–60.25)	
Risk class, *n* (%)		
1–3	11/12 (91.67%)	
4	1/12 (8.33%)	

COPD, chronic obstructive pulmonary disease.

As shown in **Table 3**, on admission, patients 5 and 7 had decreased absolute counts and percentages of lymphocytes, patients 2 and 4 had slightly increased absolute counts and percentages of neutrophils, patients 5 and 10 had slightly increased levels of C-reactive protein (CRP) and hypersensitive C-reactive proteins, and four patients had an increased erythrocyte sedimentation rate (ESR). Procalcitonin (PCT) levels and white blood cell count (WBC) was slightly increased in two patients. The levels of total protein and albumin decreased in patients 5 and 6, respectively. Levels of serum creatinine (CR) decreased in six patients and levels of blood urea nitrogen (BUN) increased in three patients. Except for patient 11, who had increased levels of alanine aminotransferase (ALT) and aspartate aminotransferase (AST) induced by anti-tuberculosis drugs, all patients had normal ALT and AST levels. CD3^+^, CD4^+^, and CD8^+^ T-cell counts were available for eight patients and patients 4, 5, and 7 had reduced CD3^+^, CD4^+^, and CD8^+^ T-cell counts, respectively.

**Table 3:** Laboratory findings on admission.

Characteristics	Patients, *n* (%)	Normal range	Unit
Routine blood test			
White blood cell count (> 9.5)	2/12 (16.67%)	3.5–9.5	10^9^/L
Neutrophil count (> 6.3)	2/12 (16.67%)	1.8–6.3	10^9^/L
Neutrophil percentage (> 75)	4/12 (33.33%)	40–75	%
Lymphocyte count(< 1.1)	5/12 (41.67%)	1.1–3.2	10^9^/L
Lymphocyte percentage (< 20)	7/12 (58.33%)	20–50	%
Monocyte count (> 0.6)	3/12 (25%)	0.1–0.6	10^9^/L
Monocyte percentage (> 10)	3/12 (25%)	3–10	%
Number of red blood cells(> 5.8 or < 4.3)	2/12 (16.67%)	4.3–5.8	10^12^/L
Hemoglobin (> 150 or < 115)	2/12 (16.67%)	115–150	g/L
Platelet distribution width (< 15.5)	7/12 (58.33%)	15.5–18.0	fL
Thrombocytocrit (< 0.11)	3/12 (25%)	0.11–0.28	%
Blood biochemistry			
Erythrocyte sedimentation rate (> 20)	5/12 (41.67%)	0–20	mm/h
Fibrinogen (> 3.5)	4/12 (33.33%)	1.8–3.5	g/L
Prothrombin time (> 14.5)	3/12 (25%)	11–14.5	s
Activated partial thromboplastin time (> 43.5)	3/12 (25%)	31.5–43.5	s
Blood potassium (> 5.1 or < 3.5)	1/12 (8.33%)	3.5–5.1	mmol/L
Blood sodium (< 137)	3/12 (25%)	137–145	mmol/L
Blood chlorine (< 99)	2/12 (16.67%)	99–110	mmol/L
Blood calcium (> 2.92 or < 2.12)	3/12 (25%)	2.12–2.92	mmol/L
Total protein (< 65)	5/12 (41.67%)	65–85	g/L
Albumin (< 40)	6/12 (50%)	40–55	g/L
Total bilirubin (> 28 or < 5.1)	1/12 (8.33%)	5.1–28	μmol/L
Gamma-glutamyltransferase (> 28)	2/12 (16.67%)	7–45	U/L
Alanine transaminase (> 40)	1/12 (8.33%)	7–40	U/L
Aspartate aminotransferase (> 40 or < 15)	2/12 (16.67%)	15–40	U/L
Lactate dehydrogenase (> 250 or < 120)	1/12 (8.33%)	120–250	U/L
Lactate (> 3.96 or < 1.32)	1/12 (8.33%)	1.32–3.96	mmol/L
Creatine kinase (< 38)	2/10 (25%)	38–174	IU/L
Creatinine (< 62)	6/12 (50%)	62–102	μmol/L
Blood urea nitrogen (> 6.1)	3/12 (37.5%)	2.5–6.1	umol/L
Pro-B-type natriuretic peptide (> 125)	4/12 (50%)	0.00–125.00	pg/ml
Blood glucose (> 5.9)	3/12 (43%)	4.1–5.9	mmol/L
Tissue polypeptide antigen (> 75)	5/11 (0.5)	0–75	U/L
Immunoglobulin E (> 100)	3/9 (42.86%)	0–100	IU/mL
C-reactive protein (≥ 10)	5/12 (36.36%)	< 10	mg/L
Hypersensitive C-reactive protein (≥ 1)	10/12 (81.82%)	< 1	mg/L
Procalcitonin (> 0.05)	2/11 (20%)	0–0.05	ng/mL
T-cell subpopulation			
CD3^+^ T-cell counts (< 770)	5/8 (57%)	770–2860	/μL
CD3^+^CD4^+^ T cells (< 500)	7/8 (86%)	500–1440	/μL
CD3^+^CD8^+^ T cells (< 238)	4/8 (43%)	238–1250	/μL
CD4/CD8(> 2.47 or < 1.00)	1/8 (14%)	1.00–2.47	

IU, international unit.

### Radiological features of the participants

Consistent with previous reports, multiple nodules (found in 11 out of 12 patients), bronchiectasis (found in 10 out of 12 patients) and cavitary opacities (found in 6 out of 12 patients) (**Supplement Figure 1**) were the radiologic features most patients had in common were the major patterns found. In addition, pulmonary consolidation was present in two patients (i.e., patients 2 and 8; **Supplement Figure 1G** and **Supplement Figure 1J**). The bronchiectasis was usually mild or moderate, multifocal, within the focal lesion correlated with localized fibrosis traction, and with no obvious peripheral exudation except for in patient 2, who had a history of bronchiectasis in which the lesion was diffuse and extensive. Each lobe, in each lung, could be involved. All the bronchiectasis were cylindrical, and mucus plugging was seen in 6 out of 10 bronchiectasis patients. After anti-NTM treatment, resolution of mucus plugging was found, but bronchiectasis was not ameliorated.

The infectious species found were *M. kansasii* (4), *M. abscessus* (3), *M. intracellulare* (2), *M. fortuitum* (2), and *M. avium* (1). The cavitary opacity is usually localized in the upper lobes, and the cavity is generally small with a thick wall or even occasionally with an air–fluid level. The species which most often had a CT presentation of cavitary opacity was *M. kansasii*. Among the six patients presenting with cavitary opacity, three were infected by *M. kansasii*, one was infected by *M. avium*, one by *M. intracellulare*, and one by *M. abscessus*. The multiple nodules were characterized with centrilobular distribution, occasionally with a tree-in-bud appearance, which appeared with or without the concomitant presence of bronchiectasis or cavity. The infectious species were *M. kansasii* (by which four patients were infected), *M. abscessus* (by which three patients were infected), *M. avium* (by which one patient were infected), *M. intracellulare* (by which two patients were infected), and *M. fortuitum* (by which two patients were infected). Usually, CT appearances of bronchiectasis, cavity and multiple nodules presented simultaneously, progressed slowly, and absorbed slowly usually with residual lung lesions after treatment.

### Conventional *microbiological tests results*

On admission, routine microbiology tests for common pathogens of community-acquired pneumonia (CAP) and *M. tuberculosis* (MTB), including serology, sputum smear and culture, and blood culture, were performed. Only patient 12’s sputum culture had the positive result for the test indicating the presence of *P. aeruginosa*. None of the patients had a positive result for the sputum smear test.

Among the 10 patients who took the serum antibody test for MTB, only one tested positive for the presence of immunoglobin M (IgM), and three tested positive for the presence of immunoglobulin G (IgG). None of the patients had a positive result for the BALF Xpert assay. Among the 12 patients who took the purified protein derivative test (PPD) for tuberculosis, patients 1, 4, and 5 were mildly (+), moderately (++) and severely (+++) positive, respectively. Among the 10 patients who took the interferon gamma release assay (IGRA), four patients were positive. None of the patients had a positive result for the serum (1–3)-β-D-glucan (G test) assay, or for the serum and BALF galactomannan (GM) assay (**Table 4**).

**Table 4:** Conventional microbiological tests.

No.	Culture	Sputum Smear	The respiratory pathogen panel by PCR	The respiratory pathogen panel by immunological test	G test	GM test	Xpert MTB/RIF	TB-Ab IgM	TB-Ab lgG	PPD^a^	IGRA
P1	Negative	Negative	NA	NA	NA	NA	NA	NA	NA	+	NA
P2	BALF: *Actinomyces dentocariosus*	Negative	NA	NA	Neg	Neg	Negative	Negative	Negative	+	+
P3	Negative	Negative	NA	NA	Neg	Neg	Negative	Negative	Negative	Negative	+
P4	Negative	Negative	NA	NA	Neg	Neg	Negative	Negative	+	++	Negative
P5	Negative	Negative	NA	NA	Neg	Neg	Negative	Negative	Negative	++	+
P6	Negative	Negative	NA	NA	Neg	Neg	Negative	Negative	Negative	+	Negative
P7	Negative	Negative	NA	NA	Neg	Neg	NA	Negative	Negative	++	Negative
P8	Negative	Negative	NA	NA	Neg	Neg	Negative	Negative	Negative	+	Negative
P9	Negative	Negative	Negative	Negative	Neg	Neg	Negative	+	+	+	+
P10	Sputum: *Pseudomonas aeruginosa*	Negative	Negative	Negative	Neg	Neg	Negative	Negative	Negative	++	Negative
P11	Negative	Negative	Negative	Negative	Neg	Neg	Negative	Negative	Negative	+++	Negative
P12	Negative	Negative	Negative	Negative	Neg	Neg	Negative	NA	NA	Negative	Negative

G test, 1,3-β-D-glucan test; GM test, galactomannan test; TB-Ab IgM, human tuberculosis antibody IgM test; TB-Ab IgG, human tuberculosis antibody IgG test; PPD, purified protein derivative test for tuberculosis; IGRA, interferon-gamma release Assays.

a: Reaction was observed between 48-72 hours for maximum induration size and results were interpreted as: 0-5 mm negative, 5-10 mm mildly

positive +, 10-20 mm moderately positive ++, more than 20 mm strongly positive +++.

As conventional microbiological testing could not provide valuable clues for the diagnosis, and the adequate duration of empiric antibiotic therapy was ineffective, BALF (11 patients) and sputum (patient 7, who had a history of anesthesia-related allergy) samples were collected for mNGS.

**Figure 1:**
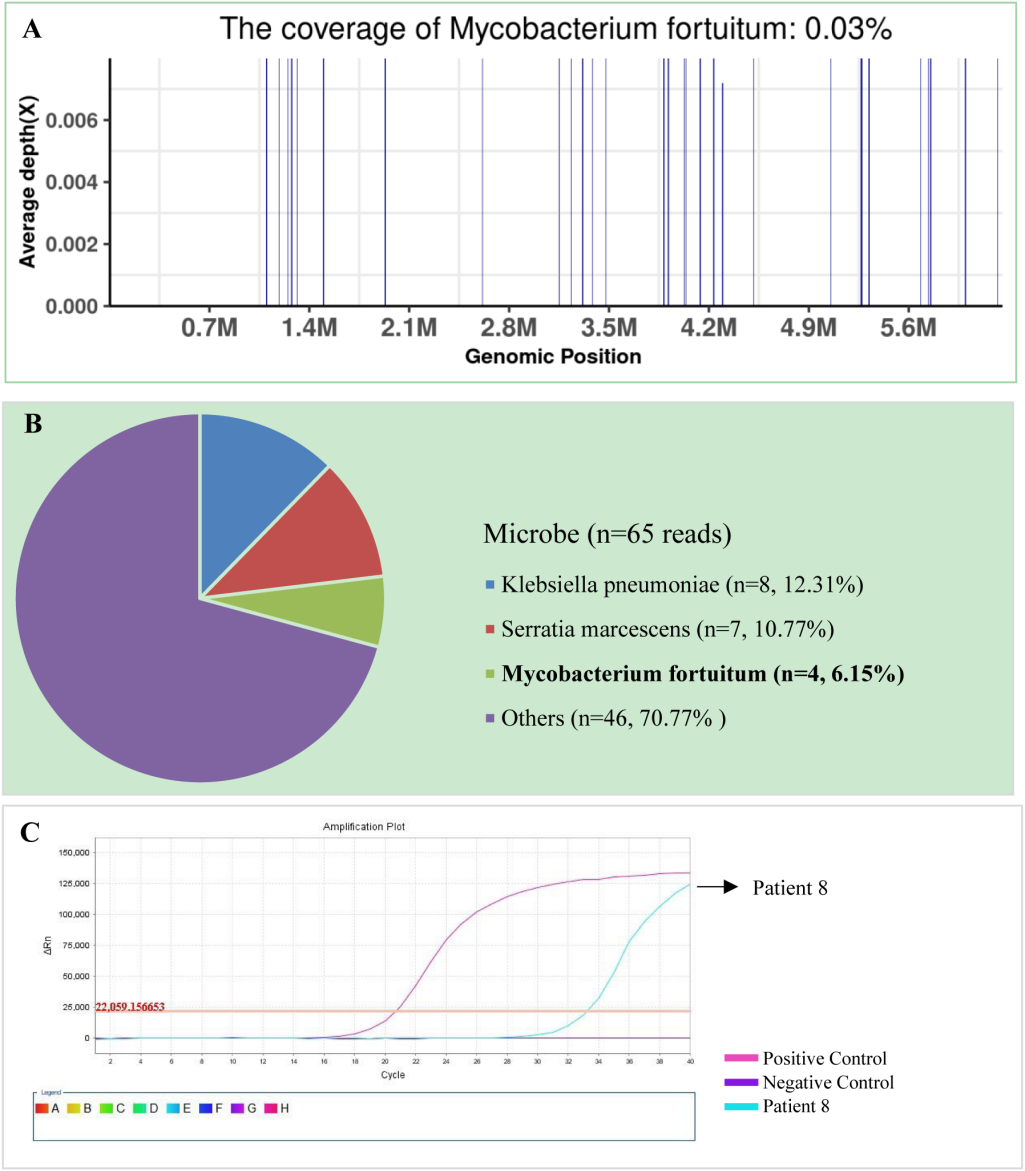
The bronchoalveolar lavage fluid (BALF), metagenomic next-generation sequencing (mNGS), and quantitative polymerase chain reaction (PCR) results for patient 8. **(A)** The genome coverage of *Mycobacterium fortuitum*, with 30 reads mapped to the genome of *M. fortuitum*. **(B)** The species composition of the BALF microbes, with four species-specific reads mapped to the genome of *M. fortuitum*. **(C)** Positive qPCR results for *M. fortuitum*.

**Figure 2:**
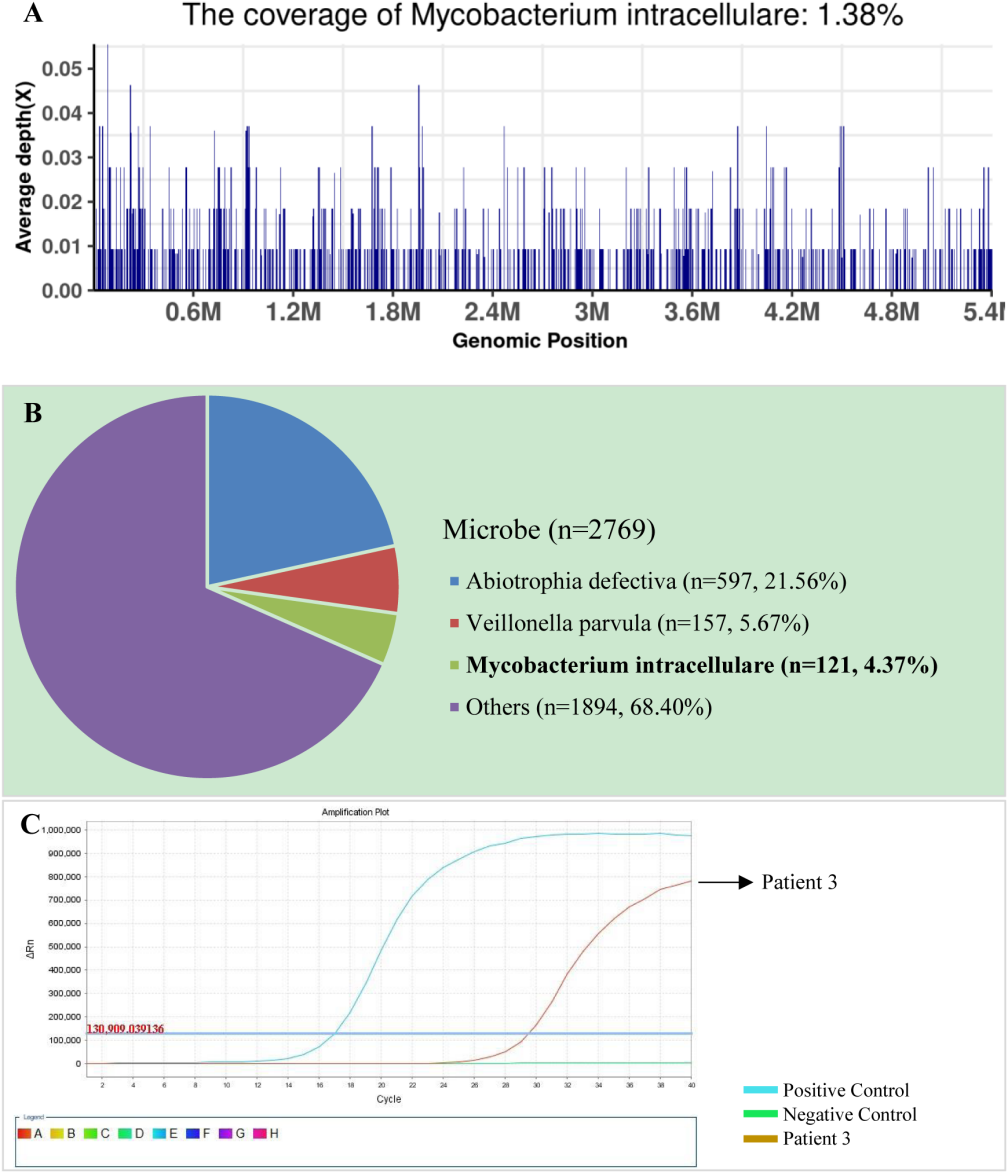
The bronchoalveolar lavage fluid (BALF), metagenomic next-generation sequencing (mNGS), and quantitative polymerase chain reaction (PCR) results for patient 3. **(A)** The genome coverage of *Mycobacterium intracellulare*, with 812 reads mapped to the genome of *M. intracellulare*. **(B)** The species composition of the BALF microbes, with 121 species-specific reads mapped to the genome of *M. intracellulare*. **(C)** Positive qPCR results for *M. intracellulare*.

The SDSMRN of non-tuberculous mycobacteria were detected in all samples. Based on Chinese guidelines, and the interpretation of mNGS results, six patients were diagnosed with NTM pulmonary diseases by BALF mNGS; these patients comprised one who had been infected by *M. avium*, one infected by *M. intracellulare*, one infected by *M. kansasii*, two infected by *M. fortuitum*, and one infected by *M. abscessus*. The SDSMRN of non-tuberculous mycobacteria, with which raw sequencing data were normalized to 20M reads, ranged from 19 to 766. Another 6 patients were diagnosed as suspected NTM pulmonary diseases due to the small number of nontuberculous mycobacteria reads (lower than 10 SDSMRN) with medium confidence. Another 6 patients were diagnosed as suspected NTM pulmonary diseases due to the small number of nontuberculous mycobacteria reads (lower than 10 SDSMRN) with medium confidence. Among them, the small unique reads of *M. intracellulare* (BALF), *M. abscessus* (*sputum*) and *M. kansasii* (BALF) were found in 1, 2 and 3 patients, respectively. qPCR was subsequently used to validate the results of mNGS in nine patients (**Figures 1**, **2**; **Figures S2–8**). Six BALF samples from patients 2, 3, 4, 8, 10, and 12 were detected to be positive (**Figure 2**; **Figures S3, S4, S6** and **S7**). Interestingly, the qPCR result of the BALF sample from patient 8 was positive, with four SDSMRNs of the *M. fortuitum* detected by an mNGS test (**Figure 2**). The mNGS and qPCR results are shown in **Table 5**.

**Table 5:** mNGS and PCR results.

Patient	Sample	Platform	NTM	SDSMRN	Other pathogens detected by mNGS (SSRN)	Turnaround time of mNGS detection	Time point of NGS test after admission	qPCR results
Patient 1	BALF	NextSeq 550Dx sequencing platform	*Mycobacterium kansasii*	1	Human betaherpesvirus *7* (11)	2 days	3 days	Negative
Patient 2	BALF	NextSeq 550Dx sequencing platform	*Mycobacterium avium*	119	None	2 days	18 days	Positive
Patient 3	BALF	NextSeq 550Dx sequencing platform	*Mycobacterium intracellulare*	104	*Tropheryma whipplei* (11)	2 days	6 days	Positive
Patient 4	BALF	NextSeq 550Dx sequencing platform	*Mycobacterium intracellulare*	5	*Enterobacter cloacae* complex (5)	2 days	1 day	Positive
Patient 5	BALF	NextSeq 550Dx sequencing platform	*Mycobacterium kansasii*	3	Human betaherpesvirus 4 (32)	2 days	15 days	Negative
Patient 6	BALF	Ion Torrent proton sequencer	*Mycobacterium kansasii*	542	*Rhinovirus A* (235871)	3 days	2 days	None
Patient 7	Sputum	NextSeq 550Dx sequencing platform	*Mycobacterium abscessus*	1	*Streptococcus pneumoniae* (6339); *Haemophilus influenza* (331); human betaherpesvirus 4 (288); and human betaherpesvirus 7 (56)	3 days	9 days	None
Patient 8	BALF	NextSeq 550Dx sequencing platform	*Mycobacterium fortuitum*	19	None	4 days	16 days	Positive
Patient 9	BALF	BGISeq-50/MGISeq-2000 platform	*Mycobacterium kansasii*	1	*Enterococcus faecium* (11); human betaherpesvirus 7 (1); and *Schizophyllum commune* (6)	4 days	4 days	None
Patient 10	BALF	NextSeq 550Dx sequencing platform	*Mycobacterium abscessus*	766	*Pseudomonas aeruginosa* (5580); and *Candida albicans* (4)	1 day	5 days	Positive
Patient 11	BALF	NextSeq 550Dx sequencing platform	*Mycobacterium abscessus*	5	Human betaherpesvirus 4 (3)	1 day	8 days	Negative
Patient 12	BALF	NextSeq 550Dx sequencing platform	*Mycobacterium fortuitum*	37	*Aspergillus flavus* (11); and *Pneumocystis jirovecii* (13)	1 day	6 days	Positive

BALF, bronchoalveolar lavage fluid.

### Treatment and *outcome*

**Figure 3:**
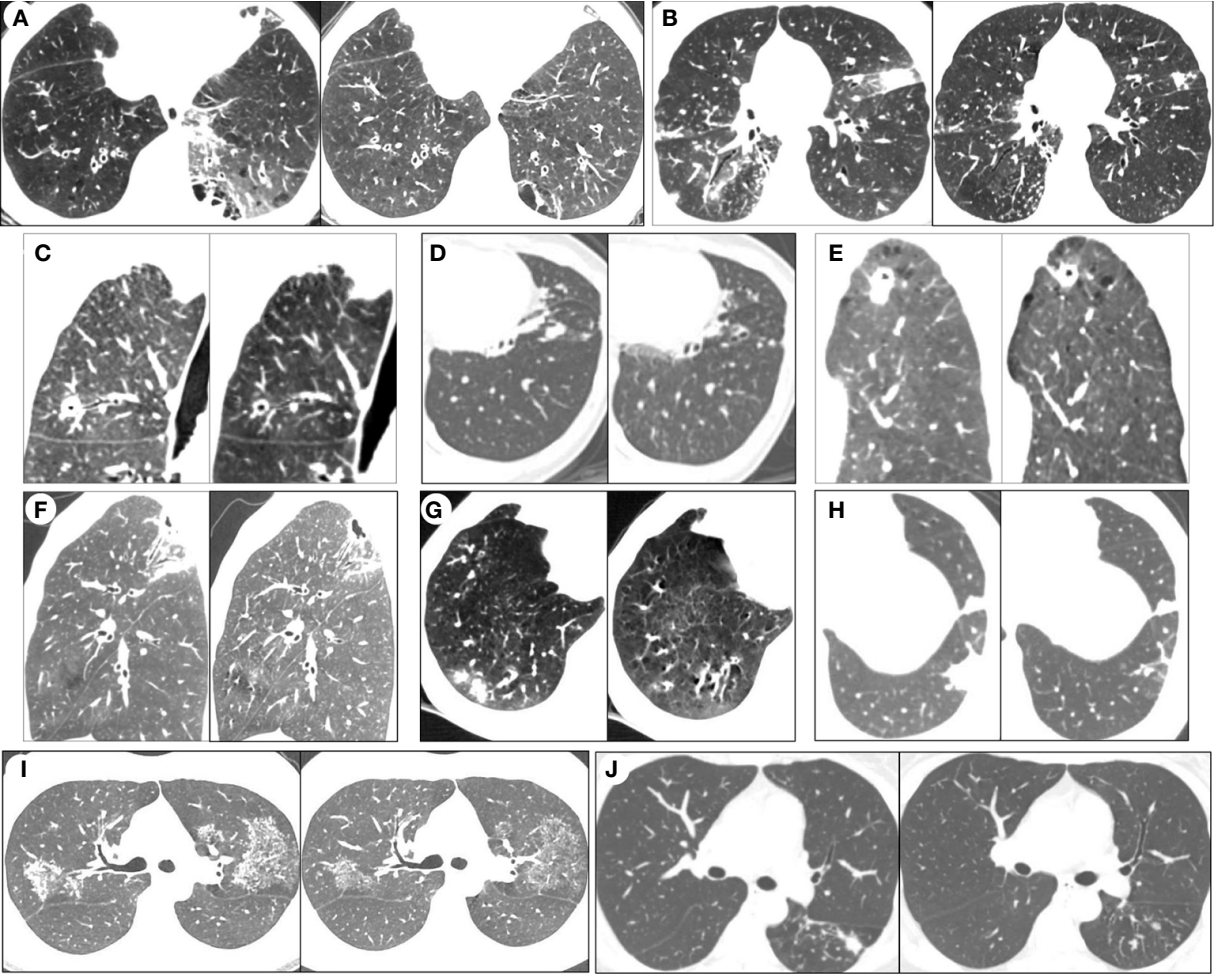
Serial chest computed tomography (CT) scans of selected patients with NTM-PD before and after the anti-NTM treatment. After 2 to 3 months of anti-NTM treatment, HRCT imaging showed significant absorption of lesions in patient 1 **(A)**, patient 2 **(B)**, patient 3 **(C)**, patient 4 **(D)**, patient 5 **(E)**, patient 6 **(F)**, patient 7 **(G)**, 464 patient 8 **(H)**, patient 9 **(I)** and patient 11 **(J)**.

On admission, all patients, except patient 11, were treated with β-lactam/β-lactamase inhibitor combinations and/or quinolones. Patient 11 was given isoniazid, rifampicin, ethambutol, and pyrazinamide because they had been diagnosed with pulmonary tuberculosis at a local hospital. When mNGS results were obtained, their treatments were adjusted to antimicrobial therapy for NTM-PD according to the ATS/ERS/ESCMID/IDSA guideline (Daley et al., 2020b) (**Table 6**). All patients, except patient 12, were discharged 1-11 days after anti-NTM therapy, with their symptoms either alleviated significantly or diminishing. Obviously, for patient 12, her symptoms were also alleviated, but she was discharged 2 months after receiving anti-NTM therapy because of her dermatomyositis. As treatments for NTM-PD must be administered for prolonged periods, all patients, except patient 10, are still in the follow-up phase. Until now (after about 2–3 months of anti-NTM treatment), among the remaining 11 patients, the lesions detectable on CT imaging show significant absorption (**Figure 3**).

**Table 6:** Treatment and outcomes.

Patient	Antimicrobial therapy before NTM diagnosis	Antimicrobial therapy after NTM diagnosis	Outcome
Patient 1	Piperacillin/tazobactam	Isoniazid + ethambutol + rifapentine	Symptoms disappeared
Patient 2	Piperacillin/tazobactam; then moxifloxacin + meropenem	Azithromycin + moxifloxacin	Symptoms alleviated obviously
Patient 3	Piperacillin/tazobactam + levofloxacin	Azithromycin + rifampicin + ethambutol	Symptoms alleviated obviously
Patient 4	Levofloxacin	Azithromycin + rifampicin + ethambutol	Symptoms alleviated
Patient 5	Piperacillin/tazobactam	Isoniazid + rifampicin + ethambutol	Symptoms alleviated obviously
Patient 6	Moxifloxacin	Isoniazid + rifampicin + ethambutol	Symptoms disappeared
Patient 7	Biapenem	Azithromycin + faropenem + amikacin	Symptoms alleviated obviously
Patient 8	Piperacillin/tazobactam + moxifloxacin	Moxifloxacin + minocycline + sulfamethoxazole	Symptoms disappeared
Patient 9	Levofloxacin	Isoniazid + rifampicin + ethambutol	Symptoms alleviated obviously
Patient 10	Piperacillin/tazobactam + moxifloxacin	Azithromycin + faropenem + amikacin	Symptoms disappeared
Patient 11	Isoniazid + rifampicin + ethambutol + pyrazinamide	Azithromycin + faropenem + amikacin	Symptoms alleviated obviously
Patient 12	Cephalosporins + moxifloxacin	Sulfamethoxazole + voriconazole + moxifloxacin	Symptoms alleviated obviously

## Discussion

Our retrospective study is the first to systematically evaluate the clinical performance and effectiveness of mNGS in the diagnosis of NTM-PD out of its referral institution. In our study, 12 patients who responded poorly to initial empiric antibiotic therapy were diagnosed with NTM-PD by way of mNGS, and given the recommended drug based on the isolated bacterial species by which they were infected. Symptoms disappeared or were subsequently alleviated in all the patients. In addition, CT imaging showed significant absorption of the lesions. In our study, the turnaround time of mNGS detection ranged from 1 day to 4 days, and was therefore significantly shorter than the turnaround time for the traditional NTM culturing process. Our result indicates that mNGS testing is an effective tool for the rapid identification of NTM. This approach could guide earlier and more targeted treatment and, finally, improve the prognoses of patients with NTM-PD.

According to the ATS/ERS/ESCMID/IDSA clinical practice guidelines for the treatment of non-tuberculous mycobacterial pulmonary disease in 2020 (Daley et al., 2020a), the main microbiological evidence of NTM pulmonary disease is positive culture results from respiratory samples or positive acid-fast staining smears or granulomatous inflammation from lung biopsies. Released in the same year, but a little later than the ATS/ERS/ESCMID/IDSA clinical practice guidelines, the Chinese guidelines added nucleic acid detection from bronchial washes or lavage as microbiological evidence of the presence of NTM (Chinese Society of Tuberculosis, 2021). Mycobacterial culturing was not routinely performed in our non-referral hospitals, both because it is time-consuming of mycobacterial culture and because of poor clinician awareness of NTM-PD. Therefore, we chose the Chinese guidelines to diagnose NTM pulmonary disease, and six patients were definitively diagnosed with NTM-PD. Owing to the difficulty of DNA extraction for NTM, we considered the mNGS result as positive when SDSMRN was ≥ 1. Meanwhile, because NTM may be due to environmental or hospital contamination, we considered a SDSMRN of ≥ 10 as the credible value for the NTM infection; this was based on a consensus in the suggestions of Chinese experts (Chinese Society of Bacterial Infection and Drug Resistance Prevention, 2022). Considering clinical manifestations, radiological imaging, and good response to anti-NTM treatment in combination, we regarded patients as having a confirmed case of NTM pulmonary disease when the SDSMRN was ≥ 10 (Chinese Society of Bacterial Infection and Drug Resistance Prevention, 2022) and as suspected NTM pulmonary disease when the SDSMRN was ≥ 1.

Several studies have validated the diagnostic performance of mNGS in the NTM pulmonary diseases. 281 One study (Xu et al., 2022) stated that mNGS and acid-fast staining was positive in 23 and 6 NTM 282 samples, showing 100% and 26.08% sensitivity, separately. In our study, all 12 NTM samples can be identified by using mNGS with 100% sensitivity, too. However, all acid-fast staining results were negative with 0% sensitivity. Another study (Wei et al., 2022) compared the performance of mNGS and Bactec mycobacterial growth indicator tubes (MGIT) 960 in NTM-PD diagnosis and found that the sensitivity of mNGS in NTM-PD diagnosis was 81.4%. The sensitivity of MGIT 906 in NTM-PD diagnosis was 53.6%, and the mNGS and MGIT 960 used in combination in NTM-PD diagnosis was 91.8%. Above all, these results indicate that the performance of mNGS in the diagnosis of NTM-PD was much better than acid-fast staining and bacterial culturing, but a little lower than the combined methods. Therefore, mNGS may be used complementarily with acid-fast staining and bacterial culturing in the diagnosis of NTM-PD, and this combined use of mNGS, acid-fast staining, and bacterial culturing may largely improve the diagnostic efficiency in the future. Besides NTM, mNGS also detected the presence of other common pathogens causing respiratory infections, such as *P. aeruginosa* in patient 10, the presence of which was confirmed in the sputum sample.

Differentiation between NTM infection and tuberculosis (TB) is important but difficult. Although recognized radiological presentations of bronchiectasis, cavitary opacities, and multiple nodules are common indicators of NTM-PD, it is difficult to diagnose NTM-PD based solely on these clinical manifestations and imaging findings, since considerable radiological overlap exists between NTM-PD and pulmonary tuberculosis (PTB) (Musaddaq and Cleverley, 2020). Usually, the diagnosis of NTM-PD is rare in the majority of hospitals without mycobacterial reference laboratories, owing to the lack of clinician awareness and poor access to adequate laboratory resources for NTM species identification or speciation (Sharma and Upadhyay, 2020), especially in high-TB-burden countries and areas, where acid-fast bacilli sputum smear tests are the primary method for the diagnosis of PTB (Gopinath and Singh, 2010). There is a high possibility of misdiagnosis of NTM-PD, as PTB diagnosis using anti-acid staining smear tests is not able to differentiate NTM from the *M. tuberculosis* complex, and these patients are empirically treated with anti-TB drugs (Gopalaswamy et al., 2020). Usually, NTM species are resistant to first-line anti-TB drugs, thus, this misdiagnosis will result in inappropriately prolonged, and potentially toxic treatment and poor outcomes (Shao et al., 2015). In our study, 10 out of 12 patients and 4 out of 10 patients were positive for the PPD test and IGRA, respectively, which are important methods used in the diagnosis of TB. It has been reported that the accuracy measures of PPD are often confounded by the bacillus Calmette–Guérin (BCG) vaccination and non-tuberculous mycobacterial (NTM) infections (Sharma et al., 2017). Although it has been claimed that IGRA is more specific than PPD in the diagnosis of TB (Ji et al., 2017), a few NTM species like *M. kansasii* could also induce a positive result for IGRA (Jagielski et al., 2019); in our study, two of the four patients with positive IGRAs were infected by *M. kansasii*. In addition, it has been reported recently that IGRA possesses limited discriminatory power for the detection of NTM in AFB smear-negative patients (Yang et al., 2020). Finally, we could not exclude the possibility of co-infection of TB and NTM in some of these patients, especially in patients 2 and 3, who were infected with *M. avium* and *M. intracellulare*, which have not been reported to induce a positive IGRA result.

In this study, qPCR was applied to validate the results of mNGS in nine patients, and only six samples were detected to be positive. Several articles have also indicated that the positive rate of mNGS was higher than qPCR in the ability to detect specific pathogens; for example, the Torque teno virus in children with leukemia (Leijonhufvud et al., 2022) and *Orientia tsutsugamushi* (Liu et al., 2021). Besides the positive rate, mNGS also exhibited several other advantages over qPCR in the detection of infectious diseases. First, the discovery power of mNGS was higher than that of qPCR. qPCR can only detect sequences of known pathogens, which were highly suspected by the clinics. In contrast, mNGS is a hypothesis-free approach capable of identifying almost all microbes (DNA and RNA viruses, parasites, fungi, and bacteria) in samples and does not need prior sequence information knowledge, making it especially beneficial for the etiological diagnosis of rare and critical diseases (Ramachandran and Wilson, 2020). Second, mNGS can be characterized as being high throughput. A single mNGS experiment can detect more than one clinical specimen. Using the Illumina NextSeq 550 DX sequencer as an example, which was the mainstream sequencer in the industry of mNGS, a single run can generate more than 400 million reads per run and, thus, can detect as high as 20 samples in parallel (Diao et al., 2022). Meanwhile, a single qPCR experiment can only detect one sample. Therefore, mNGS is suitable for pathogen identification in rare, critical, and difficult-to-detect diseases.

Bronchiectasis is often seen in NTM-PD patients. Previous studies have shown that the incidence of NTM-PD in bronchiectasis patients was significantly higher than in those without bronchiectasis (Aksamit et al., 2017). Furthermore, pre-existing bronchiectasis was deemed as an important risk factor for the occurrence of NTM-PD (Yang et al., 2021). Meanwhile, patients with NTM-PD can also develop bronchiectasis because of repeated infections, which can cause permanent inflammation and scarring of the airways (Chalmers and Sethi, 2017). In our study, we found that only one patient with evidence of bronchiectasis on their chest CT scan had a prior history of bronchiectasis, and for the remaining patients, the CT imaging of bronchiectasis did not ameliorate after treatment (although other patterns of abnormalities showed significant absorption), and this further supported the possible role of NTM infection in causing bronchiectasis directly. Furthermore, pathological findings of bronchiectasis, including bronchial cartilage and smooth muscles layer destruction, airway obstruction by granulomas, and bronchial mucosa ulceration, have been observed in patients diagnosed with NTM-PD(Fujita et al., 2003). In addition, given the generally relative low virulence and slow growth (Ratnatunga et al., 2020), NTM was generally considered to be of low pathogenicity to humans (van Ingen, 2013), the chronic infection was usually accompanied with fibrosis, which could dilate the bronchi with its mechanical traction; this was in accordance with the presence of multifocal bronchiectasis within the focal lesion with little peripheral exudation in our study. So, for patients without prior history of bronchiectasis, NTM infection might be a cause of bronchiectasis.

Our results demonstrated that acute infectious indices, such as WBC count and PCT, were nearly normal, whereas the inflammatory indices including NE percentage, CRP, and ESR were moderately increased in some patients. This, to some degree, provides additional evidence of the relatively low virulence and slow growth of NTM. However, other factors including comorbidities, such as the acute exacerbation of COPD, autoimmune diseases, and use of glucocorticoids and immunosuppressants, can also affect the infectious and inflammatory indices. In addition, lymphocytes, CD8^+^ T-cell counts, and, especially, CD4^+^ T-cell counts, were found to be low in patients with NTM-PD, indicating the impaired immunity of these patients. Another noticeable feature was the low BMI in these patients, which was in consistent with previous studies (Song et al., 2021). A lower BMI indicated the poor nutritional status of patients, which was a risk factor for the development of NTM-PD, and was correlated with poor progression (Kim et al., 2017). Thus, clinicians should pay attention to the nutritional status of individuals with predisposing factors for NTM-PD.

There are several limitations to our study. First, because of the retrospective study design, and the fact that our study was performed in a non-referral institution for NTM-PD, only one patient had a positive mycobacterial culture result; thus, a direct comparison of clinical performance between mNGS and culture was not available. Second, several laboratory tests were not taken or missed in the patients’ medical record, including standard polymerase chain reaction (PCR) panel analysis for respiratory tract pathogens, and CD4^+^ and CD8^+^ T lymphocyte tests. Third, because of the small sample size from a single center, all results were formed through descriptive analysis and the interpretation of our findings was to some degree limited.

## Data availability statement

The data presented in the study are deposited in the NCBI Sequence Read Archive database (https://www.ncbi.nlm.nih.gov/sra) with the accession number from SRR18576135 to SRR18576144, and SRR22346179.

## Ethics statement

The studies involving human participants were reviewed and approved by the Second Clinical Hospital of Chongqing Medical University. Written informed consent for participation was not required for this study in accordance with the national legislation and the institutional requirements.

## Author contributions

JL designed research. JL and XW collected data. JL and JW analyzed data. JW and HX wrote the paper. All authors contributed to the article and approved the submitted version.
