# Supplementary material for: Rapid diagnosis of non-tuberculous mycobacterial pulmonary diseases by metagenomic next-generation sequencing in non-referral hospitals
Source: Front Cell Infect Microbiol. 2023 Jan 24;12:1083497. doi: 10.3389/fcimb.2022.1083497 (PMC9902348; doi:10.3389/fcimb.2022.1083497)
Supplement: Supplementary file 1 [file DataSheet_1.docx]

**Figure S1. Representative HRCT images of patients with NTM-PD.**


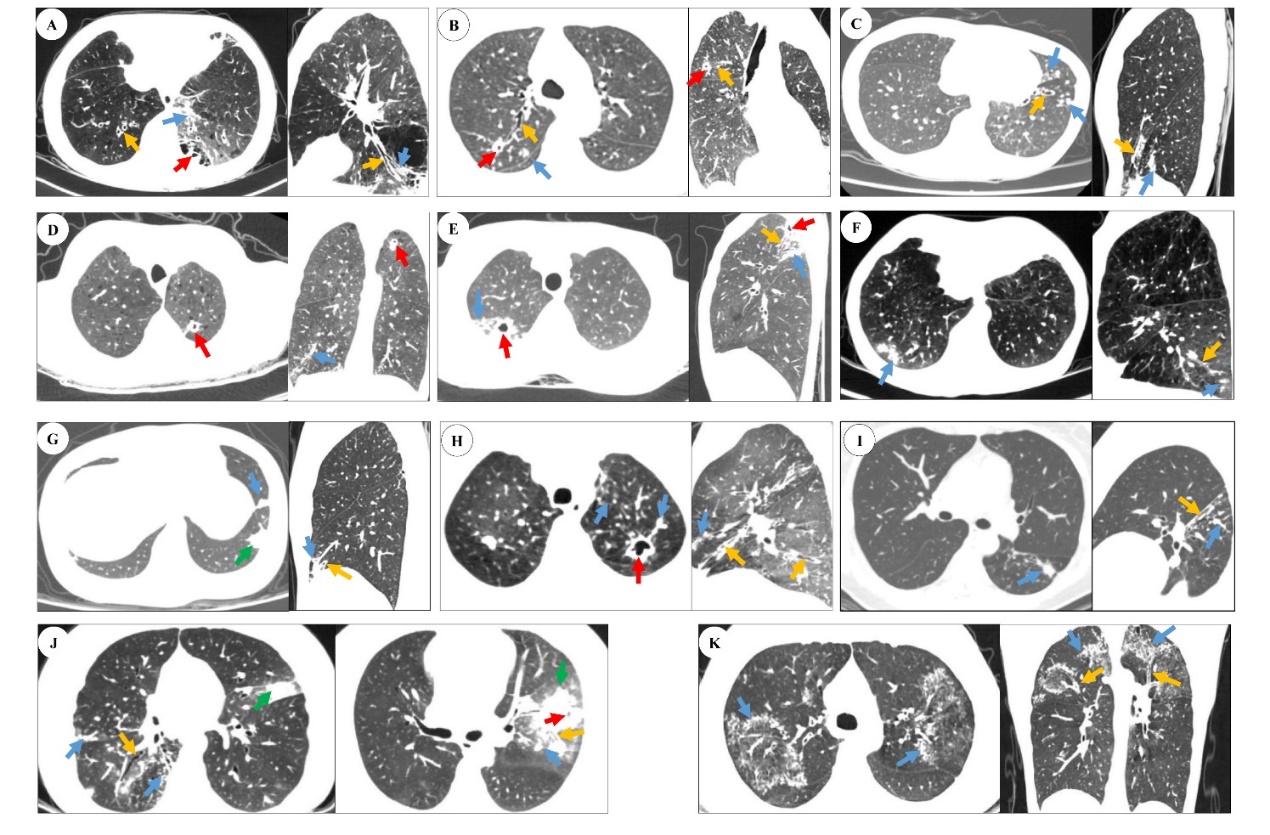


**(A)** Bilateral bronchiectasis and multiple nodules in the inferior lobes, along with a thick-walled cavity with an air-fluid level in the left inferior lobe surrounded by patchy opacity in patient 1. **(B)** Multiple nodules, focal bronchiectasis and a small thick-walled cavity in the right upper lobe in patient 3. **(C)** Multiple nodules and focal bronchiectasis in the left lingual lobe in patient 4. **(D)** A small thick-walled cavity in the left upper lobe and multiple nodules in the right inferior lobe in patient 5. **(E)** Multiple nodules, focal bronchiectasis and a small thick-walled cavity surrounded by patchy opacity in the right upper lobe in patient 6. **(F)** Multiple nodules and focal bronchiectasis in the right inferior lobe in patient 7. **(G)** Nodules, consolidation and focal bronchiectasis in the left inferior lobe in patient 8. **(H)** Clusters of multiple small nodules and focal bronchiectasis in bilateral upper lobes in patient 10. **(I)** Clusters of multiple small nodules and focal bronchiectasis in the left superior segment in patient 11. **(J)** Bilateral multiple nodules and focal bronchiectasis, consolidation with a small cavity in the left right lobe in patient 2. **(K)** Clusters of multiple small nodules and focal bronchiectasis in bilateral upper lobes in patient 9.

Blue, yellow, red and green arrow indicated multiple nodules, bronchiectasis, cavity and consolidation respectively.

**Figure S2. The BALF mNGS result of Patient 1**


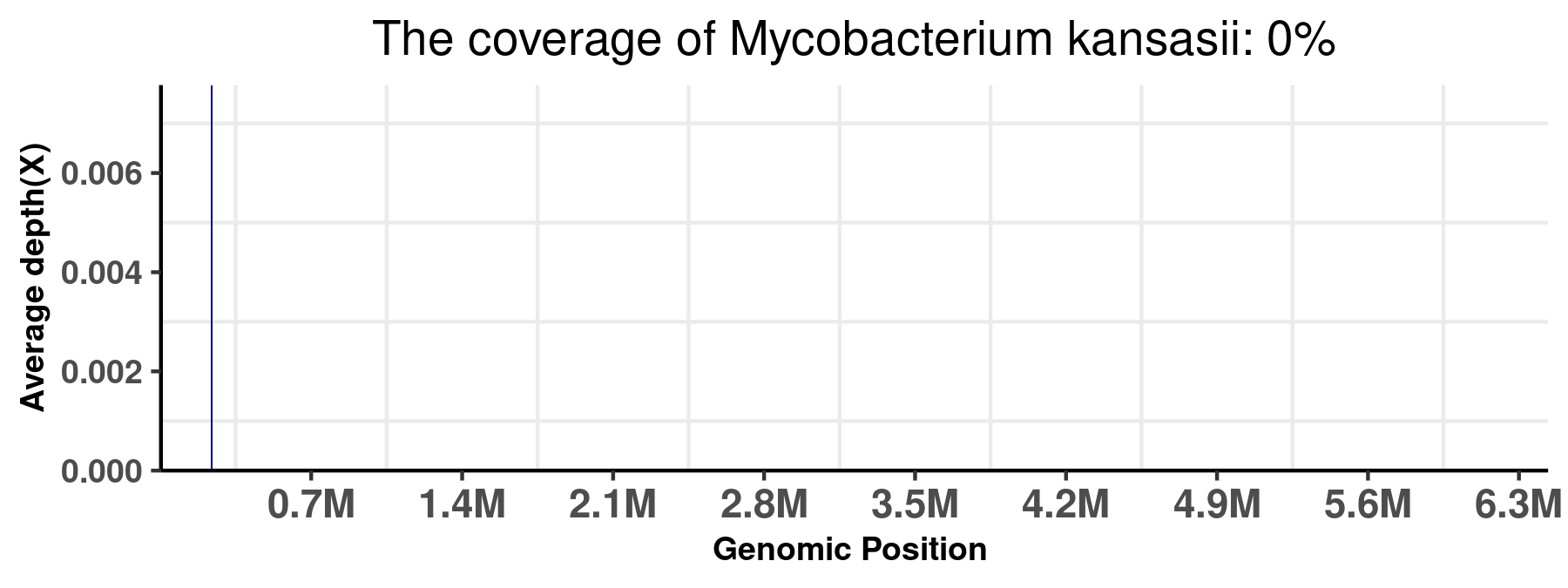


**A**

**B**

**C**

Positive Control

Negative Control

Patient 1

Patient 1


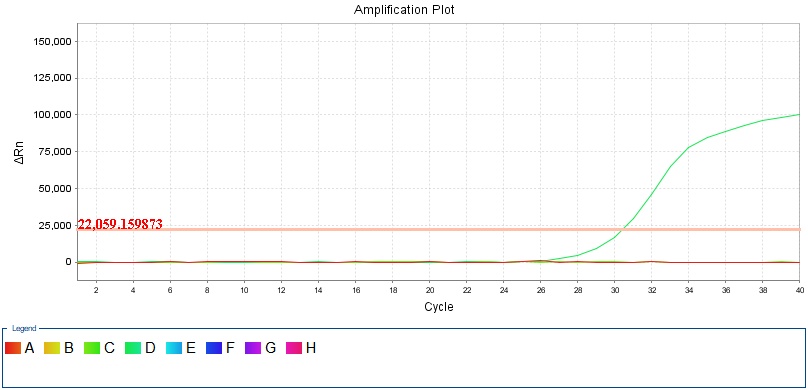


(A) The genome coverage of *Mycobacterium kansasii*, with only one read mapped to the genome of *Mycobacterium kansasii*; (B) The species composition of the BALF microbes, with one species-specific reads mapped to the genome of *Mycobacterium kansasii*; (C) Negative qPCR results of *Mycobacterium kansasii*.

**Figure S3. The BALF mNGS result of Patient 2**

**
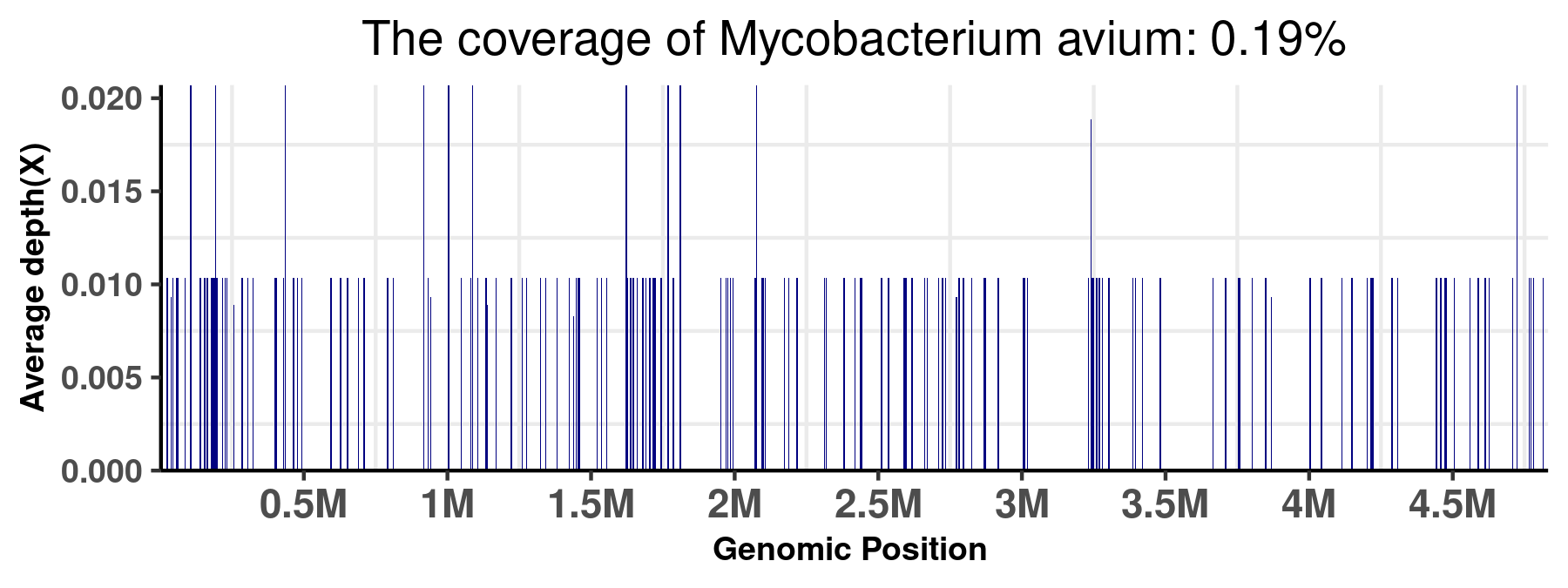
**

**A**

**B**

**C**

Positive Control

Negative Control

Patient 2

Patient 2


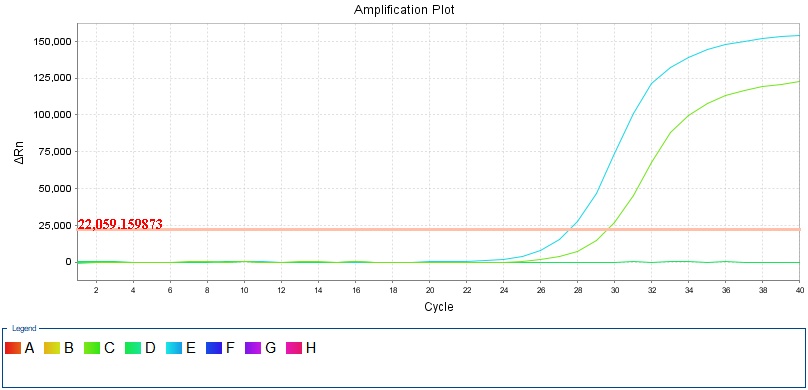


(A) The genome coverage of *Mycobacterium avium*, with 172 reads mapped to the genome of *Mycobacterium avium*; (B) The species composition of the BALF microbes, with 125 species-specific reads mapped to the genome of *Mycobacterium avium*; (C) Positive qPCR result of *Mycobacterium avium*.

**Figure S4. The BALF mNGS and qPCR result of Patient 4.**

**
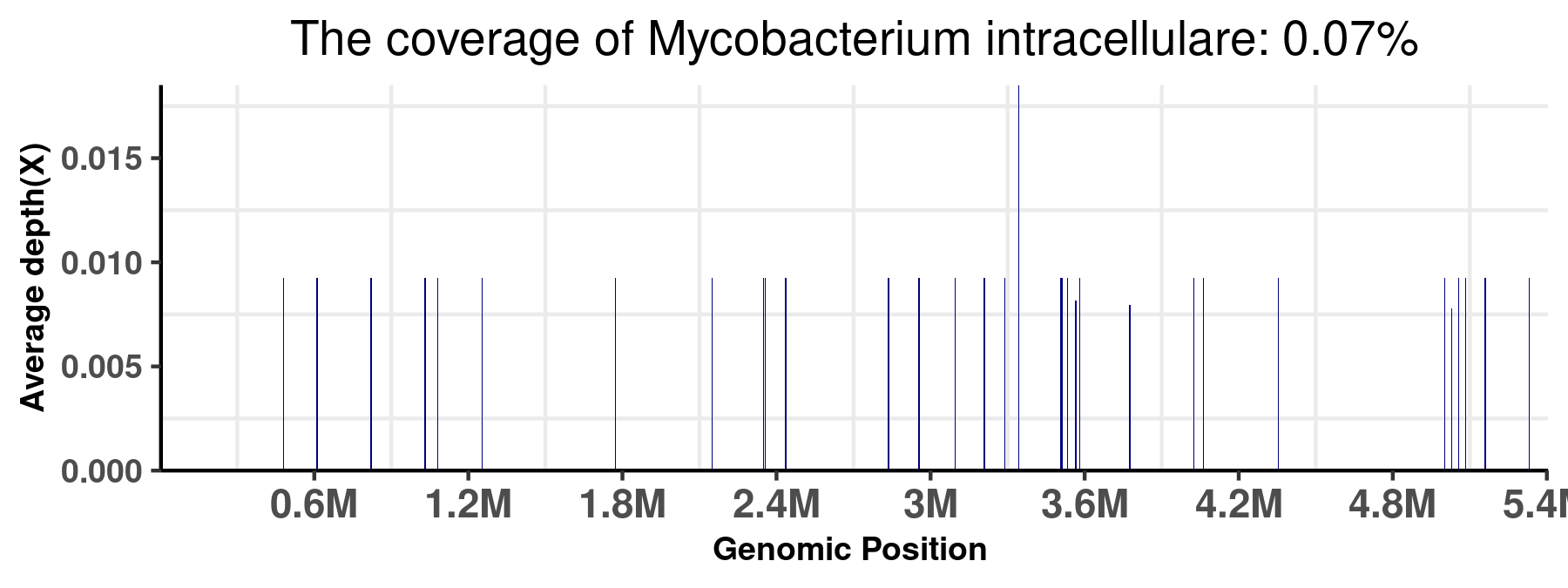
**

**A**

**B**

**C**

Patient 4

Positive Control

Negative Control

Patient 4


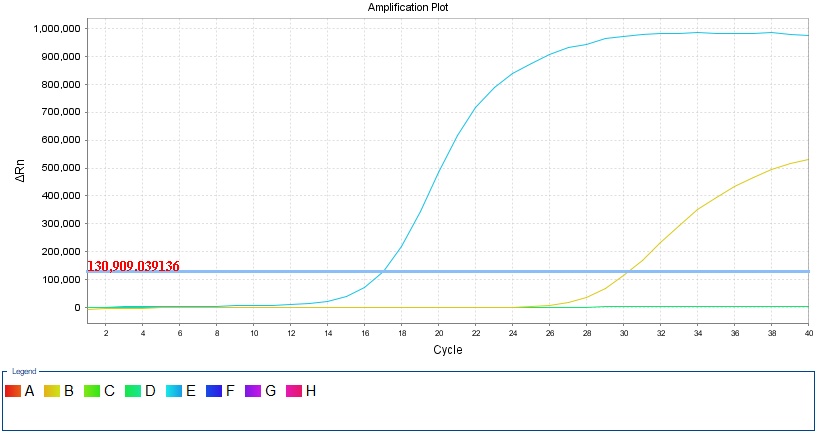


(A) The genome coverage of *Mycobacterium intracellulare*, with 33 read mapped to the genome of *Mycobacterium intracellulare*; (B) The species composition of the BALF microbes, with 5 species-specific reads mapped to the genome of *Mycobacterium intracellulare*; (C) Positive qPCR result of *Mycobacterium intracellulare*.

**Figure S5. The BALF mNGS result of Patient 5**

**
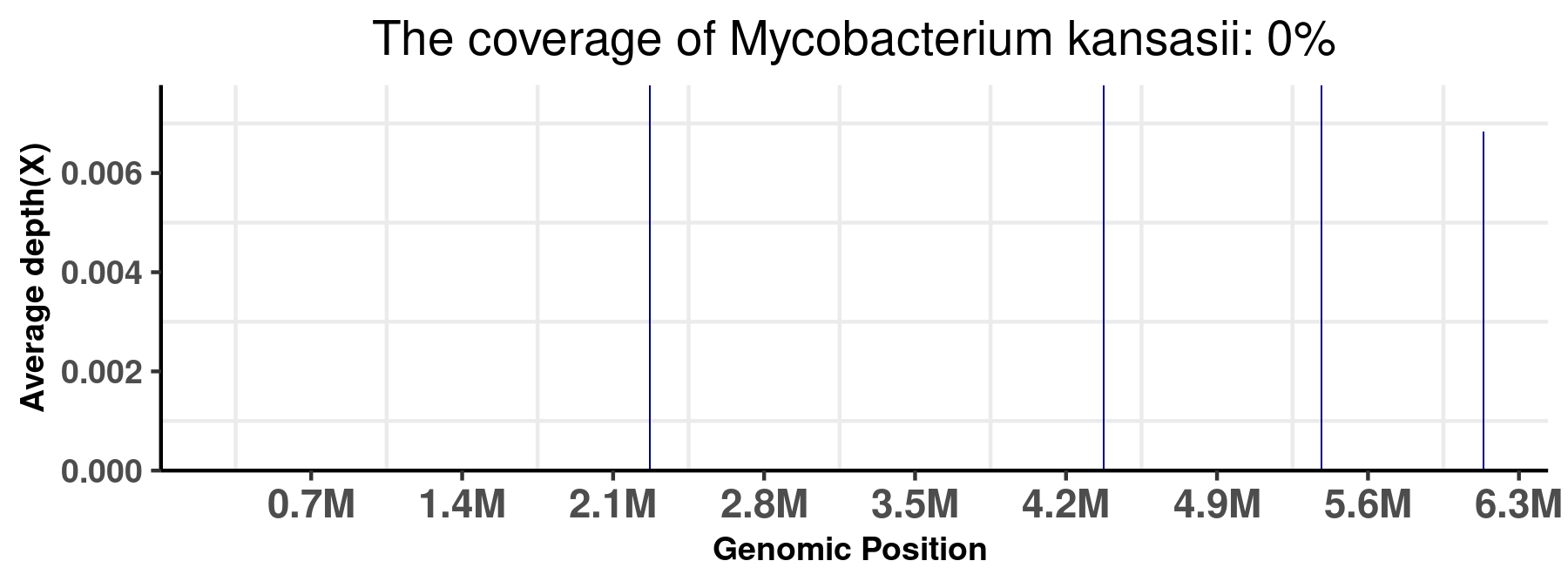
**

**A**

**B**

**C**

Positive Control

Negative Control

Patient 5

Patient 5


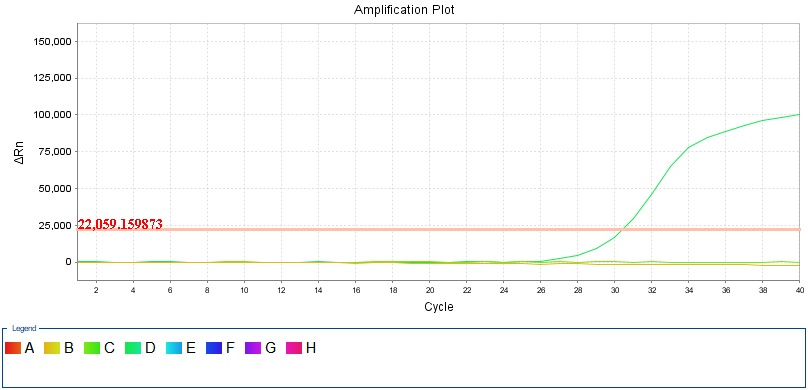


(A) The genome coverage of *Mycobacterium kansasii*, with 4 reads mapped to the genome of *Mycobacterium intracellulare*; (B) The species composition of the BALF microbes, with 3 species-specific reads mapped to the genome of *Mycobacterium intracellulare*; (C) Negative qPCR result of *Mycobacterium intracellulare*.

**Figure S6. The BALF mNGS result of Patient 10**

**
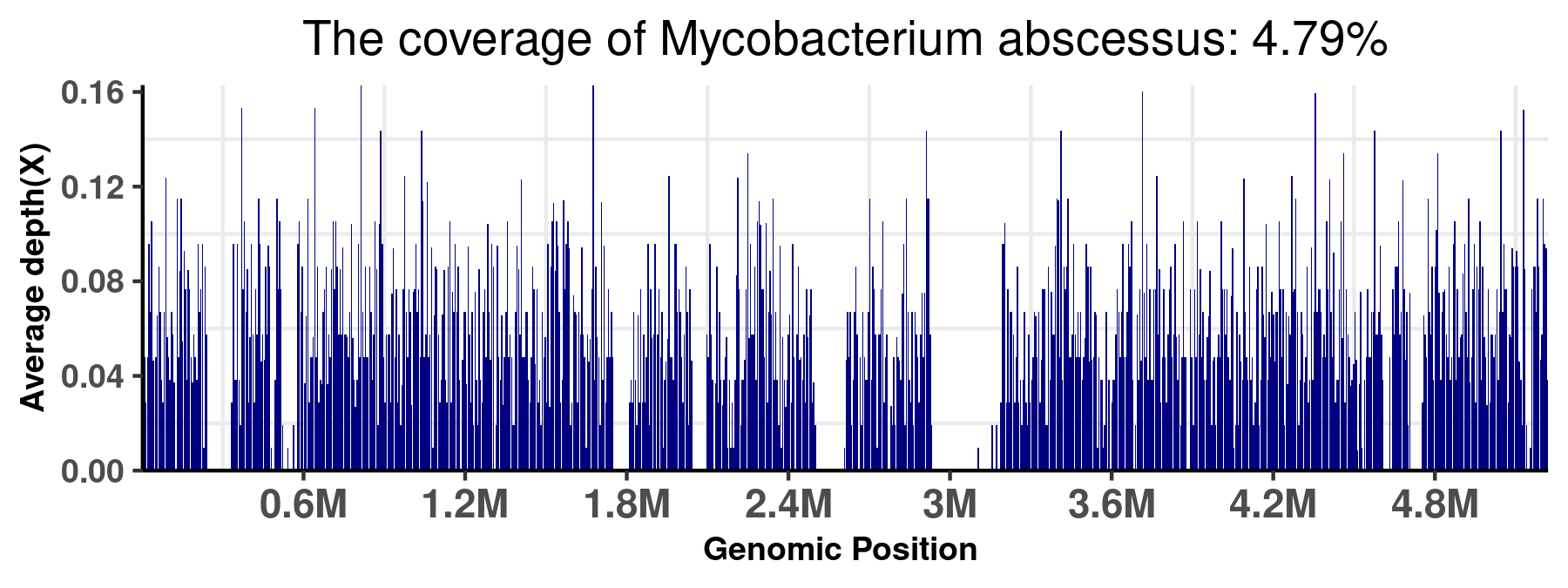
**

**A**

**B**

**C**

Positive Control

Negative Control

Patient 10

Patient 10


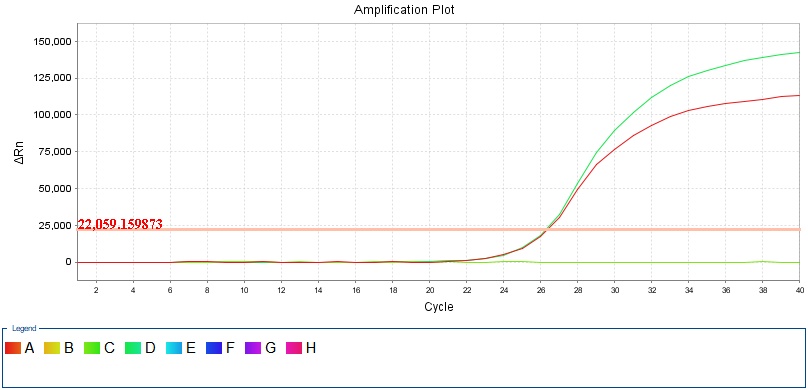


(A) The genome coverage of *Mycobacterium abscessus*, with 5, 555 reads mapped to the genome of *Mycobacterium abscessus*; (B) The species composition of the BALF microbes, with 4, 090 species-specific reads mapped to the genome of *Mycobacterium abscessus*; (C) Positive qPCR result of *Mycobacterium abscessus*.

**Figure S7. The BALF mNGS result of Patient 11**

**
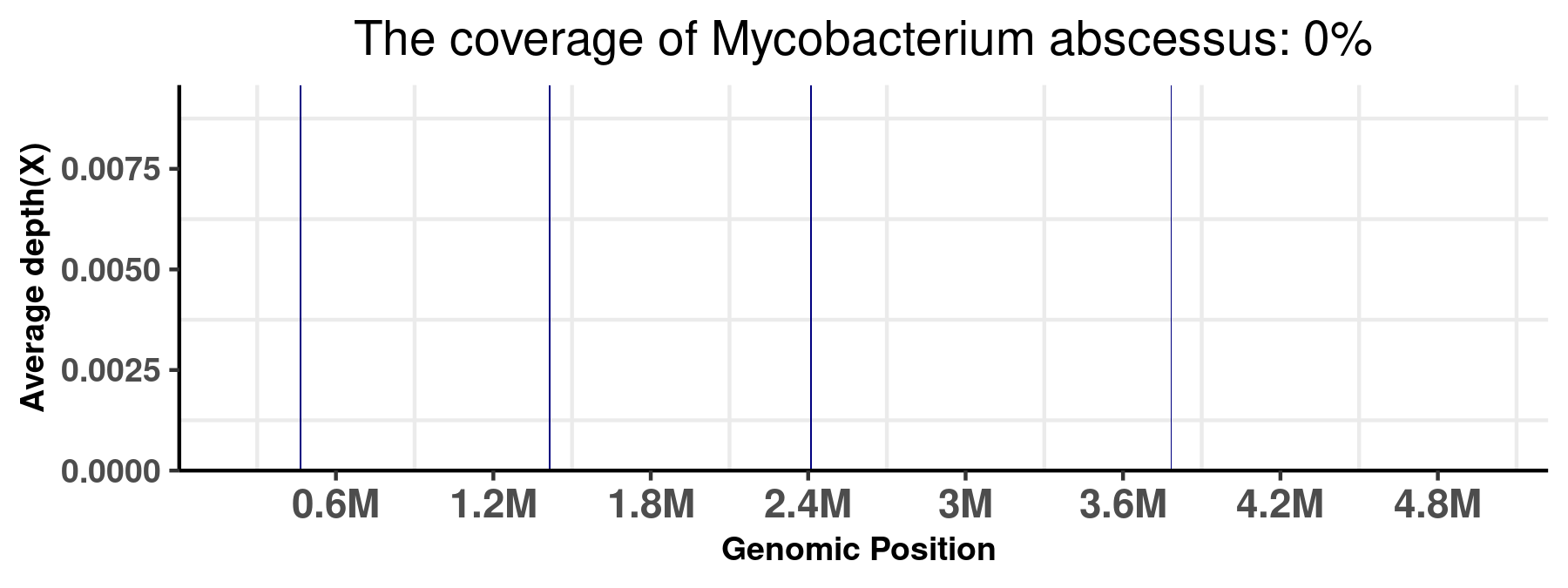
**

**A**

**B**

**C**

Patient 11

Positive Control

Negative Control

Patient 11


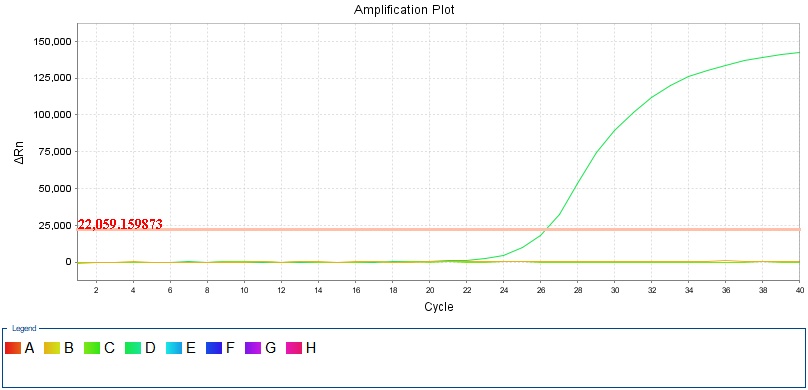


(A) The genome coverage of *Mycobacterium abscessus*, with 4 reads mapped to the genome of *Mycobacterium abscessus*; (B) The species composition of the BALF microbes, with 3 species-specific reads mapped to the genome of *Mycobacterium abscessus*; (C) Negative qPCR result of *Mycobacterium abscessus*.

**Figure S8. The BALF mNGS result of Patient 12**

**
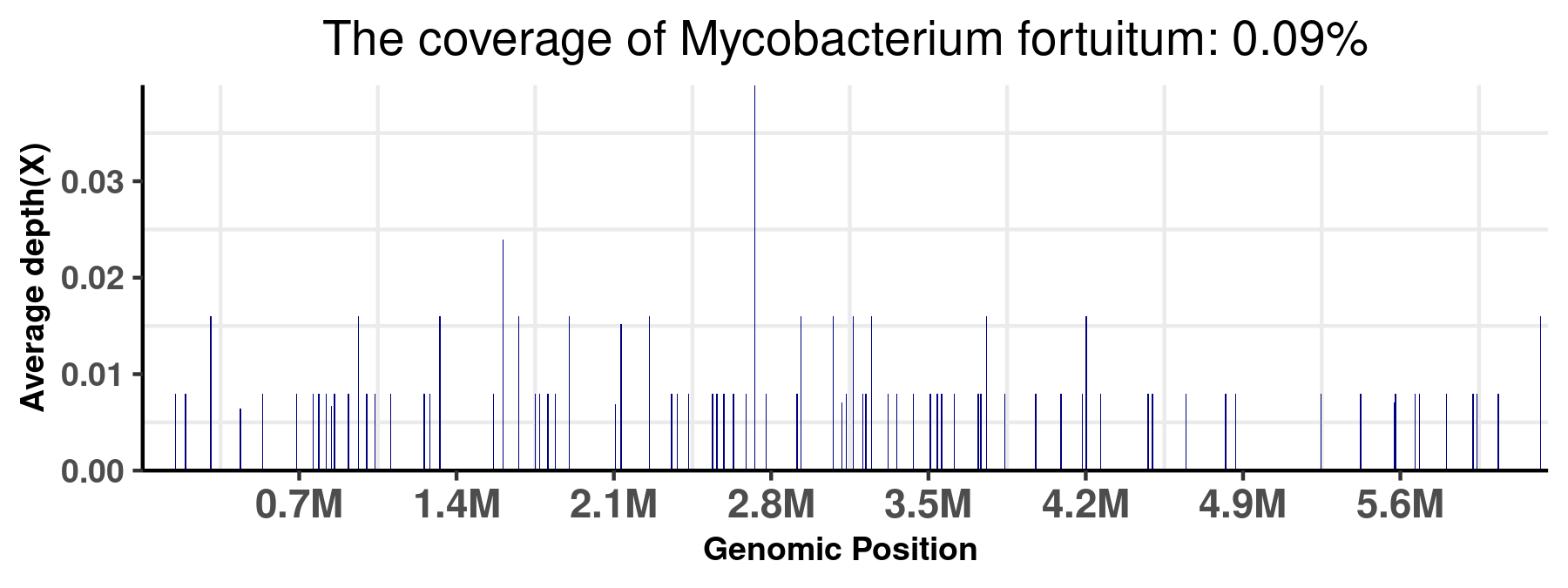
**

**A**

**B**

**C**

Positive Control

Negative Control

Patient 12

Patient 12


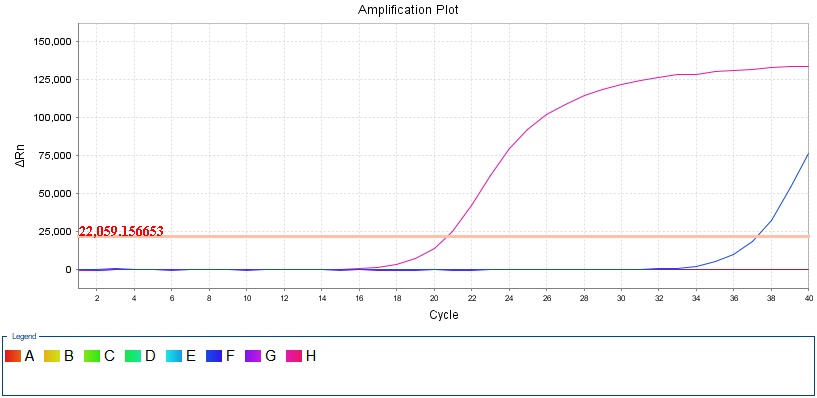


(A) The genome coverage of *Mycobacterium fortuitum*, with 101 reads mapped to the genome of *Mycobacterium fortuitum*; (B) The species composition of the BALF microbes, with 81 species-specific reads mapped to the genome of *Mycobacterium fortuitum*; (C) Positive qPCR result of *Mycobacterium fortuitum*.
